# Differential Expression of Duplicate Insulin-like Growth Factor-1 Receptors (*igf1rs*) in Medaka Gonads

**DOI:** 10.3390/life12060859

**Published:** 2022-06-08

**Authors:** Wenbo Wei, Yefei Zhu, Cancan Yuan, Yuli Zhao, Wenzong Zhou, Mingyou Li

**Affiliations:** 1 Key Laboratory of Exploration and Utilization of Aquatic Genetic Resources, Ministry of Education, Shanghai Ocean University, Shanghai 201306, China; m200100082@st.shou.edu.cn (W.W.); d180100018@st.shou.edu.cn (Y.Z.); yuancancan@yeasen.com (C.Y.); Yuli_zhao@xihuasci.com (Y.Z.); 2 Eco-Environmental Protection Research Institute, Shanghai Academy of Agricultural Sciences, 1000 Jinqi Road, Shanghai 201403, China; 3 Yeasen Biotechnology Co., Ltd., 800, Qingdai Road, Pudong New Area, Shanghai 201318, China; 4 Shanghai Xihua Scientific Co., Ltd., Building 6-118, Furonghua Road, Pudong District, Shanghai 201318, China

**Keywords:** *igf1rs*, gene expression, medaka, ovary, testis

## Abstract

Insulin-like growth factor-1 receptors (*igf1rs*) play important roles in regulating development, differentiation, and proliferation in diverse organisms. In the present study, subtypes of medaka *igf1r*, *igf1ra*, and *igf1rb* were isolated and characterized. RT-PCR results showed that *igf1ra* and *igf1rb* mRNA were expressed in all tissues and throughout embryogenesis. Using real-time PCR, the differential expression of *igf1ra* and *igf1rb* mRNA during folliculogenesis was observed. The results of in situ hybridization (ISH) revealed that both of them were expressed in ovarian follicles at different stages, and *igf1rb* was also expressed in theca cells and granulosa cells. In the testis, both *igf1ra* and *igf1rb* mRNA were highly expressed in sperm, while *igf1rb* mRNA was also obviously detected in spermatogonia. In addition, *igf1ra* mRNA was also present in Leydig cells in contrast to the distribution of *igf1rb* mRNA in Sertoli cells. Collectively, we demonstrated that differential *igf1rs* RNA expression identifies medaka meiotic germ cells and somatic cells of both sexes. These findings highlight the importance of the *igf* system in the development of fish gonads.

## 1. Introduction

The *igf* signaling system is a growth factor complex containing ligands, receptors, and binding proteins, which exists in all vertebrates [1]. The role of the *igf* signaling system in growth regulation has been well established, and its important roles in gonadal development has been gradually explored [2]. *Igf1* mRNA is expressed in spermatogonia and spermatocytes as well as Leydig cells in Nile tilapia (*Oreochromis niloticus*) [3]. *Igf2* could influence germ cell proliferation in the testis of *Hypostomus garmani* [4]. Furthermore, the gonad-specific *igf3* can regulate spermatogenesis and reproduction in teleosts, such as Nile tilapia and zebrafish (*Danio rerio*) [5,6]. *Igf2bp3* deletion leads to abnormal germ plasm assembly and a reduction in the number of germ cells in zebrafish [7]. Furthermore, *igf1r* is also expressed in the testis and ovary, which indicates the important roles of *igf1r* in gonadal development and gametogenesis [8,9].

*Igf1r* is a cell surface receptor that belongs to the tyrosine kinase receptor superfamily, which is expressed in diverse tissues of organisms [10]. In mammals, there is only one *igf1r*. *Igf1r* has been detected in sperm of humans, and its levels is positively correlated with sperm concentration [11]. Besides, *igf1r* is also expressed in ovaries of alpaca (*Vicugna pacos*), including follicles, granulosa, and theca interna cells [12]. In the cultured testicular cells, *igf1r* is highly expressed in Sertoli cells but also in spermatogonia and primary spermatocytes [13].

Due to whole genome duplication, fish have two subtypes of *igf1r*, *igf1ra* and *igf1rb* [14], and both of them play important biological functions. *Igf1ra* and *igf1rb* mRNA are highly expressed in gonads during vitellogenesis and spermatogonia proliferation in *Pampus argenteus* [15]. In adult zebrafish, *igf1ra* and *igf1rb* have distinct expression patterns, and the relative abundance of *igf1ra* and *igf1rb* is different in tissues [16]. It has been shown that inhibition of the *igf* signal pathway by knocking down *igf1rb* in the embryo of zebrafish can result in mis-migration and apoptosis of primordial germ cells (PGCs) [17]. Therefore, more attention has been focused on their cellular localization and biological activity in fish gonads. However, the role of *igf1r* in reproduction and gonadal development has rarely been studied, especially in fish.

Medaka (*Oryzias latipes*) is a good model and has been widely used in investigating developmental biology [18] and stem cell biology [19,20]. In addition, the primordial germ cells specification [21], migration [22], and sex-determination mechanism [23,24] have been systematically explored. Previously, we have investigated that *igf1* is present in meiotic germ cells and somatic cells of both sexes in medaka [25]. *Igf2* is associated with self-renewal of the embryonic stem cell [26]. *Igf3* expression occurs both in germ cells and somatic cells in the ovary [27]. In the present study, to figure out the distribution and differences between *igf1ra* and *igf1rb*, the temporal and spatial expression patterns of their mRNAs in gonads, developing embryos, and follicles at different developmental stages were investigated. The expression patterns between *igf1ra* and *igf1rb* mRNAs in the gonads were also carried out by in situ hybridization studies. Our findings highlight the potential roles of the *igf* system in the reproduction and development of medaka and teleosts.

## 2. Materials and Methods

### 2.1. Fish and Embryos

Animal experiments were conducted strictly following the requirements of the Committee for Laboratory Animal Research at Shanghai Ocean University. Medaka was maintained in glass tanks with a water temperature of 26 °C, and an automatic photoperiod of 14 h light/10 h dark cycle was set. The developmental stage of medaka embryos has been previously described [28].

### 2.2. Isolation of Ovarian Follicles

The developing stages of the ovary were determined based on the original definition as described previously [25]. The ovaries were dissected from the anesthetized female medaka. As follicles have different stages, the same developmental stages were manually collected together and used for subsequent experiments: primary growth (stage I, below 0.1 mm in diameter), pre-vitellogenic (stage II, about 0.30 mm), early vitellogenic (stage III, about 0.40 mm), mid-vitellogenic (stage IV, about 0.50 mm), and fully-grown (stage V, about 0.65 mm).

### 2.3. RNA Extraction

Total RNA of adult tissues and embryos at different developmental stages of medaka was extracted by a TRIzol reagent (Invitrogen, Carlsbad, CA, USA). The NanoDrop 2000 Spectrophotometer (Thermo Fisher Scientific, Waltham, MA, USA) was used to detect the RNA quality and quantity. Furthermore, the integrity of RNA was verified by 1% agarose gel stained with nucleic acid dyes. Then, the cDNA was synthesized according to M-MLV reverse transcriptase (Takara, Shiga, Kusatsu, Japan) with an oligo(dT) _18_ primer.

### 2.4. Cloning and Sequence Analysis of Medaka Igf1r

By searching the NCBI Gene database (https://www.ncbi.nlm.nih.gov/gene/ accessed on 1 March 2020), two computational predicted cDNAs encoding medaka *igf1ra* (Gene ID: 101173298) and *igf1rb* (Gene ID: 101163560) were obtained, respectively. To verify the accuracy of the two sequences, the Open Reading Frame (ORF) of *igf1ra* and *igf1rb* was cloned and sequenced. Afterwards, the *igf1ra* and *igf1rb* putative proteins were aligned with the *igf1r* orthologs from other examined organisms by using Vector NTI Advance 11.5 software (Thermo Fisher Scientific). The phylogenetic tree was based on the MEGA X program with the neighbor-joining (NJ) method [29]. All the primers used in the present study are listed in Table 1.

### 2.5. RT-PCR and Real-Time PCR Analysis

The expression of *igf1r* isoforms was detected by RT-PCR amplification using the primers in Table 1, and *β*-actin was used for calibration. PCR was performed for 35 cycles, and the reaction system contained 1 μL of cDNA template, 12.5 μL of 5 U/μL Premix Taq (EX Taq version) (Takara), and 0.5 μL of 10 mM each of the forward and reverse primers, and deionized water was added to replenish the total volume to 25 μL. The reaction procedures were as follows: 95 °C for 10 s, annealing at 58 °C for 10 s, and extension at 72 °C for 1 min. The PCR products were then detected on a 1.5% agarose gel stained with nucleic acid dyes and analyzed by a bio-imaging system (Bio-Rad, Hercules, CA, USA).

Real-time PCR (qPCR) was performed under the CFX96 ^TM^ Real-Time System (Bio-Rad) using the SYBR Green PCR Master Mix Kit (Takara). Using *β*-actin as calibration, the relative abundance of *igf1ra* and *igf1rb* mRNA was determined using the 2^−ΔΔct^, as described previously [30]. The data were presented as the mean ± SEM (*n* = 3). Statistical analyses were evaluated using a one-way ANOVA (*p* < 0.05) in GraphPad Prism 7 software.

### 2.6. RNA In Situ Hybridization

Sections in situ hybridization (SISH) were carried out as described previously [31]. Briefly, the gonads were fixed in 4% paraformaldehyde and then dehydrated gradiently with 20% and 30% sucrose. Next, the gonads were soaked with an embedding agent, Optimal Cutting Temperature (O.C.T., Sakura, Torrance, CA, USA), and subjected for sections with a freezing microtome (Leica, Wetzlar, Germany). To synthesize probes, the partial cDNA sequences of *igf1ra* and *igf1rb* obtained by PCR were inserted into the pGEM-T vector and sequenced for verification. The plasmids were then linearized with an appropriate restriction enzyme for the synthesis of probes by using the DIG or FITC RNA Labelling Kit (Roche, Basel, Switzerland). RNA of SISH was stained with BCIP/NBT and Fluorecent in situ hybridization (FISH) was carried out by using the (TSA ^TM^) Plus Fluorescence Systems according to the product manual (Life Technologies, Carlsbad, CA, USA). The nucleus was stained with DAPI.

### 2.7. Microscopic Observation

Microscopy was performed as described [32]. In brief, microscopic observation and micrographs of gonadal sections were taken on a Nikon Ds-Ri2 camera (Nikon, Tokyo, Japan).

## 3. Results

### 3.1. Cloning and Sequence Analysis of Medaka Igf1r

**Figure 1:**
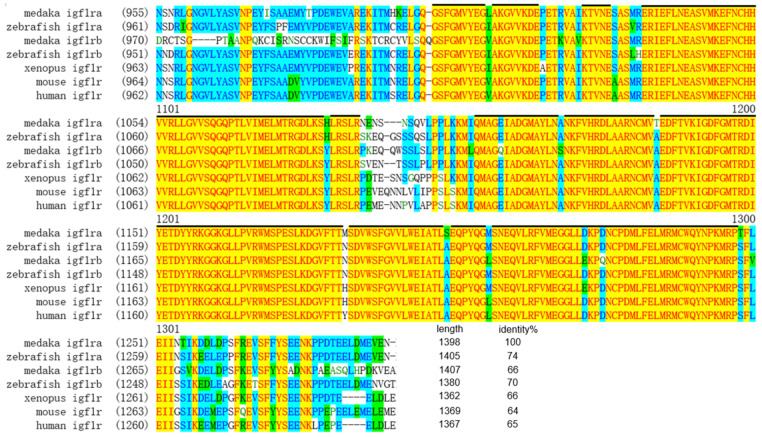
Multiple sequence alignment of medaka Igf1rs. The alignment sequences show the intracellular protein tyrosine kinase domain. Conserved regions between species are highlighted. The length and percentage identity values of Igf1rs homologs are given at the end of the alignment.

**Figure 2:**
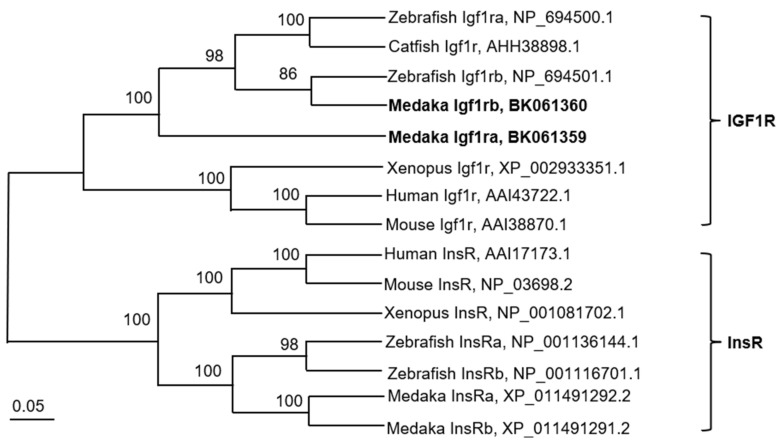
Phylogenetic tree of Igf1r. The insulin receptor (InsR) served as the out-group. Bootstrap values are given, and the bar indicates number of substitutions per site. Accession numbers are after the organism. Igf1rs from different species are clustered together, indicating that generation of *igf1r* and *insr* took place in early vertebrate evolution.

PCR was employed to amplify the sequences of *igf1r* derived from tissues and embryo samples. The *igf1ra* ORF was obtained by TA cloning, which was 4197 nt and encoded 1398 amino acid residues (GenBank accession no. BK061359) (Figure S1). However, the *igf1rb* ORF was 4224 nt for 1407 amino acid residues (GenBank accession no. BK061360) (Figure S2). The IGF1Rs’ alignment showed the intracellular protein tyrosine kinase domain on the *β*-subunit of Igf1r (Figure 1). Igf1ra protein and Igf1rb protein of medaka are highly similar to those of zebrafish, with 74% and 71% identity, respectively, according to the Igf1rs multiple sequence alignment (Figure S3). Besides, the phylogenetic tree showed that compared with medaka Igf1ra, zebrafish Igf1rb is much closer to medaka Igf1rb (Figure 2). Such a divergence was also observed for *Takifugu rubripes* and *Salmo salar* (data not shown). Furthermore, although both Igf1ra and Igf1rb existed in different regions of different chromosomes in other species, they all showed strong chromosome synteny (Figure S4).

### 3.2. RT-PCR Analysis of Igf1r RNA Expression

**Figure 3:**
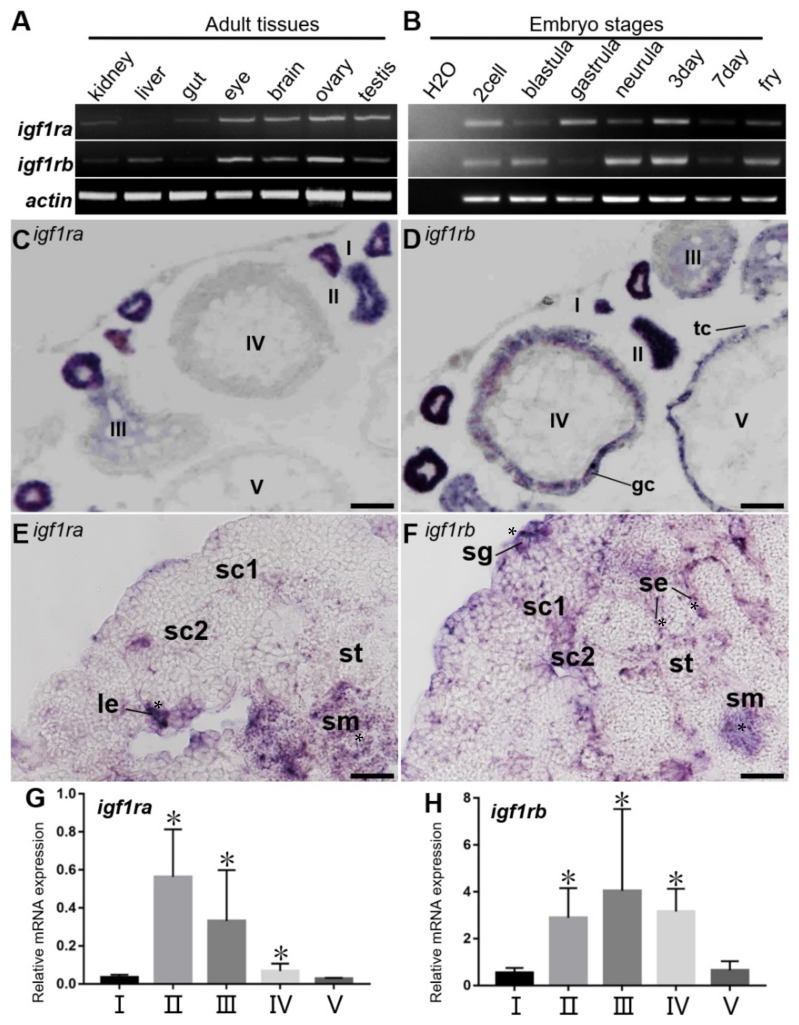
Expression of *igf1ra* RNA and *igf1rb* RNA. (**A**,**B**) RT-PCR analysis of medaka *igf1ra* and *igf1rb* in adult tissues (**A**) and developing embryos (**B**). (**C**–**F**) Ovarian and testicular cryosections using antisense *igf1ra* and *igf1rb* probes and the signals were visualized by chromogenic staining. (**G**,**H**) qPCR results of *igf1ra* and *igf1rb* at different stages of follicles. I–V, stages of oocytes; sg, spermatogonia; sc, spermatocytes; st, spermatids; sm, sperm; gc, granulosa cells; tc, theca cells; se, Sertoli cells; le, Leydig cells. Scale bars, 100 µm.

The results of the RT-PCR showed that *igf1ra* and *igf1rb* were expressed in all adult tissues and embryos. Evidently, the expression of *igf1ra* and *igf1rb* was lower in the kidney, liver, and gut, in comparison with the eye, brain, ovary, and testis (Figure 3A). Besides, these two genes were similarly expressed during embryogenesis (Figure 3B). Furthermore, qPCR was performed to further analyze the expression profiles of the two *igf1r* types at different stages of folliculogenesis. The level of *igf1ra* increased from PG (stage I) to PV (stage II) and then decreased slowly, and it was hardly expressed in FG (stage V) (Figure 3G). The expression of *igf1rb* increased from the PG stage, peaked in EV (stage III), and weakened afterward (Figure 3H). These data indicate that *igf1r* was dynamically and differentially expressed during folliculogenesis in medaka.

### 3.3. Gonadal Expression of Igf1ra RNA and Igf1rb RNA by ISH

To better understand the subcellular distributions of *igf1r*, a chromogenic SISH was performed on cryosections. In the ovary, *igf1ra* RNA and *igf1rb* RNA were found in the cytoplasm of oocytes from stages I–IV (Figure 3C,D). Furthermore, *igf1rb* was also found in theca cells and granulosa cells, while *igf1ra* was absent (Figure 3D). In the testis, the *igf1ra* mRNA was highly expressed in sperm at the later stage of spermatogenesis (Figure 3E). Remarkably, *igf1rb* mRNA was detected in spermatogonia and sperm and was weakly detected in spermatocytes as well as spermatids, which was significantly different from the expression of *igf1ra* mRNA (Figure 3F). Furthermore, a positive signal for *igf1ra* was also found around Leydig cells, whereas the signal of *igf1rb* existed around Sertoli cells (Figure 3E,F). On the contrary, both of the sense probes, as controls, got no signal above the background (data not shown).

### 3.4. Ovarian Differential Expression of Igf1ra and Igf1rb RNAs by Fluorescence ISH

**Figure 4:**
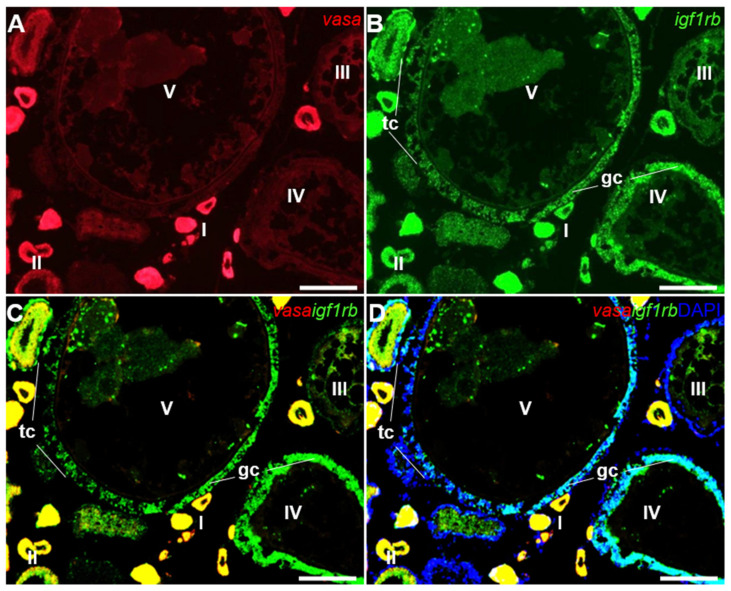
Expression of *igf1rb* RNA and *vasa* RNA in the ovary. FISH on ovarian cryosections using antisense RNA probes and the signals were visualized by fluorescence staining. The *vasa* RNA was stained in red, and the *igf1rb* was stained in green. Nuclei were stained in blue with DAPI. (**A**,**B**) Different stages of oocytes (I–V), granulosa cells and theca cells were indicated by sticks. (**C**,**D**) The merges of *vasa* with *igf1rb* and *vasa* with *igf1rb* and DAPI. I–V, stages of oocytes; gc, granulosa cells; tc, theca cells. Scale bars, 25 µm.

To further accurately identify the RNA expression of *igf1r*, the co-distribution of *igf1rb* and *vasa* was carried out by FISH. *Vasa* is a well-studied gene in medaka and other species, which is restrictively expressed in the germ cells of both sexes [33]. In the ovary, the *vasa* signal was expressed obviously in pre-meiotic oocytes and was decreased with the process of oogenesis (Figure 4A). Conversely, the *igf1rb* signal was easily found in oocytes from stages I–IV (Figure 4B). Notably, the *igf1rb* positive signal was also obvious in the granulosa cells and theca cells at later folliculogenesis (Figure 4C,D).

**Figure 5:**
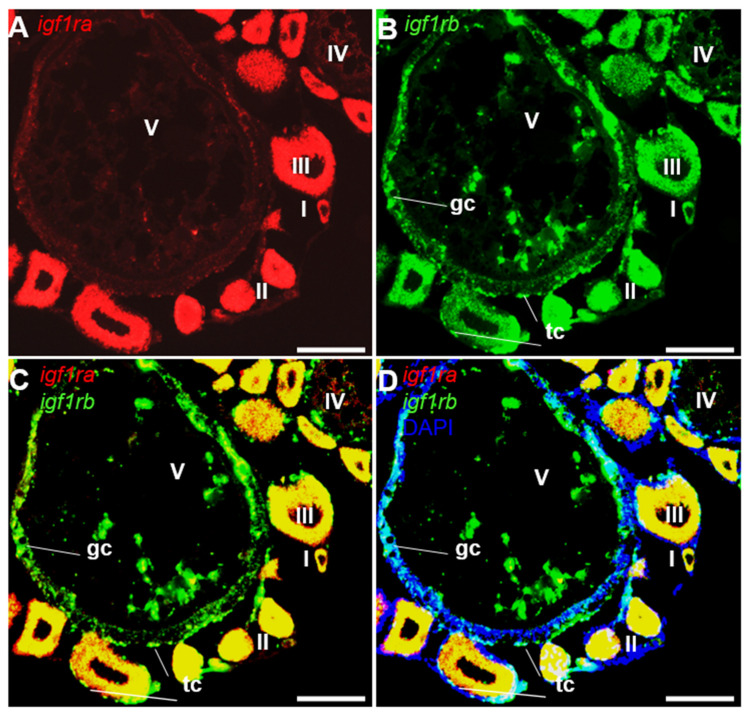
Expression of *igf1ra* RNA and *igf1rb* RNA in the ovary. FISH on ovarian cryosections using antisense RNA probes and the signals were visualized by fluorescence staining. The *igf1ra* RNA was stained in red, and the *igf1rb* was stained in green. Nuclei were stained in blue with DAPI. (**A**,**B**) Different stages of oocytes (I–V), granulosa cells and theca cells were indicated by sticks. (**C**,**D**) Merges of *igf1ra* with *igf1rb* and *igf1ra* with *igf1rb* and DAPI. I–V, stages of oocytes; gc, granulosa cells; tc, theca cells. Scale bars, 25 µm.

We compared the RNA expression of *igf1ra* with *igf1rb* by dual-color FISH. Conforming to their observations by chromogenic staining, both *igf1ra* and *igf1rb* RNAs were expressed in oocytes from stages I to IV (Figure 5A,B). Furthermore, *igf1rb* was detected abundantly in somatic cells, including granulosa cells, as well as theca cells at later developmental oocytes, while *igf1ra* was not detected in these somatic cells (Figure 5C,D)

### 3.5. Testicular Differential Expression of Igf1ra and Igf1rb RNAs by Fluorescence ISH

**Figure 6:**
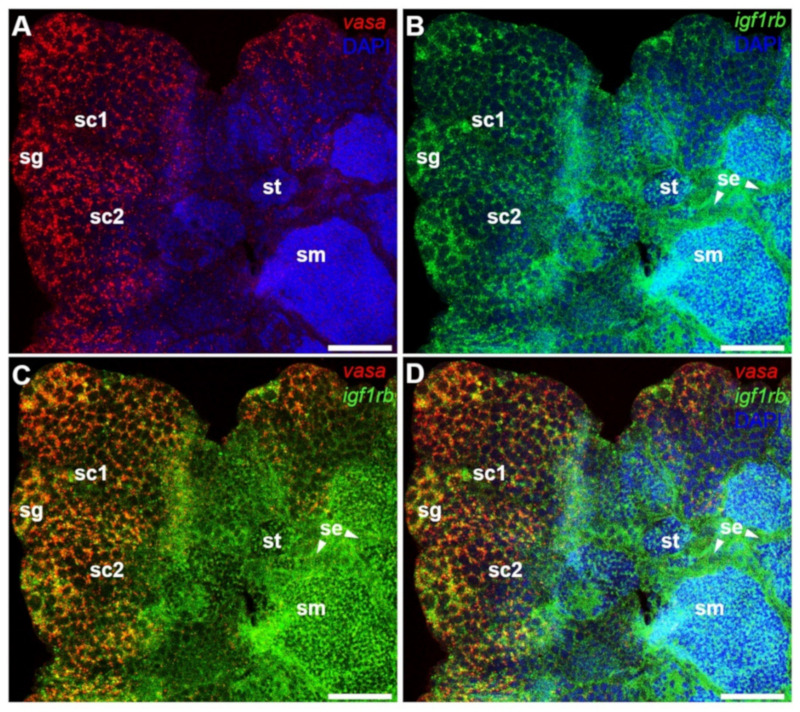
Expression of *vasa* RNA and *igf1rb* RNA in the testis. After hybridization with antisense *vasa* and *igf1rb* RNA probes, the signals were visualized by fluorescence staining. Nuclei were stained blue by DAPI. (**A**–**D**) Merges of *vasa* with DAPI, *igf1rb* with DAPI, *vasa* with *igf1rb*, and *vasa* with *igf1rb* and DAPI. Sertoli cells were indicated by arrows. *Vasa* and *igf1rb* showed significantly different expression patterns. The *vasa* signal peaked in spermatogonia and then gradually decreased until it disappeared in sperm. Conversely, the *igf1rb* was obviously detected in spermatogonia, sperm, and Sertoli cells. sg, spermatogonia; sc, spermatocytes; st, spermatids; sm, sperm; se, Sertoli cells. Scale bars, 25 µm.

In the next step, we compared the RNA expression of *igf1rb* with *vasa* by dual color FISH. In the testis, the *vasa* signal was strong in spermatogonia, and with the progress of spermatogenesis, the intensity of the *vasa* signal was decreased until it disappeared in sperm (Figure 6A). In contrast, the *igf1rb* mRNA was abundantly expressed in the spermatogonia and sperm, and it was relatively low in spermatocytes as well as in spermatids (Figure 6B). Surprisingly, a positive signal for *igf1rb* was also detected between the cyst and the cyst of the testis structure (Figure 6C,D), which is generally thought to be the location of Sertoli cells [34].

**Figure 7:**
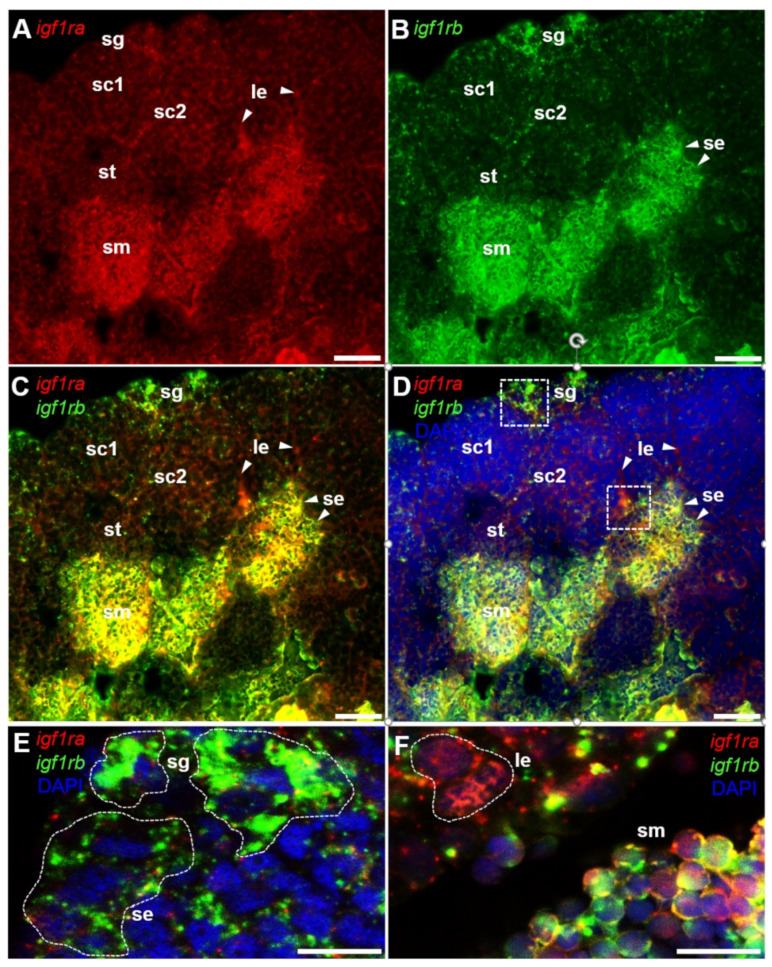
Expression of *igf1ra* RNA and *igf1rb* RNA in the testis. Adult testicular cryosections were hybridized to antisense RNA probes and the signals were visualized by fluorescence staining. Nuclei were stained blue by DAPI. (**A**,**B**) Lower magnification view showing different stages of spermatogenesis. Sertoli cells and Leydig cells were indicated by arrows. (**C**,**D**) Merges of *igf1ra* with *igf1rb* and *igf1ra* with *igf1rb* and DAPI. (**E**,**F**) Larger Magnification of panels D (white frame), highlighting the different cells. The *igf1ra* signal exists in sperm and Leydig cells. Notably, the *igf1rb* signal exists in spermatogonia, sperm, as well as Sertoli cells. se, Sertoli cells; le, Leydig cells; sm, sperm; sc, spermatocytes; sg, spermatogonia. Scale bars, 25 µm.

Then, a dual-color FISH was performed to precisely compare the expression of *igf1ra* and *igf1rb* RNA. Results indicated that *igf1ra* mRNA was richly expressed in sperm, but it was not in other stages of spermatogenic cells (Figure 7A,C). In contrast, *igf1rb* mRNA was expressed in almost all stages of spermatogenesis, especially spermatogonia and sperm. (Figure 7B–E). Furthermore, *igf1ra* mRNA and *igf1rb* mRNA were also found in somatic cells, with *igf1ra* expressed in the intertubular space where the Leydig cells usually located, while *igf1rb* mRNA delineated the germinal cysts called Sertoli cells (Figure 7A–F).

## 4. Discussion

In contrast to a single *igf1r* in mammals, many fish species have two *igf1r* paralogous isoforms, *igf1ra* and *igf1rb*, such as zebrafish [16], gilthead seabream (*Sparus aurata*) [9], and *Epinephelus coioides* [35]. According to the present study, medaka also has two *igf1r* subtypes. A sequence comparison and phylogenetic tree of the medaka two Igf1rs protein sequences with other vertebrates indicated that the receptors were highly conservative in the process of vertebrate evolution. The structure of the medaka *igf1rs* is most similar to that of zebrafish *igf1rs* [16]. Not only is their sequence identity of medaka *igf1rs* and zebrafish *igf1rs* greater than 60%, but structural motifs are nearly conserved in the two medaka *igf1rs*, such as the ATP-binding site, ligand-binding region, tyrosine kinase domain, autophosphorylation site, and IRS-I docking site. Igf1r is composed of extracellular **α**-subunits and transmembrane-spanning **β**-subunits, which contain cytoplasmic tyrosine kinase activity [36]. Activated *igf1r* phosphorylates specific reaction components, including IRS-1, IRS-2, and SRC homology collagen, and it regulates downstream responses through the phosphatidylinositol 3-kinase (PI3K)/AKT and mitogen-activated protein kinase (MAPK) pathways [1,37].

Both *igf1r* mRNAs were extensively expressed in different tissues in the adult medaka, which was consistent with reports from other teleosts [38]. The rich expression of two *igf1r* mRNAs in the eye, brain, and gonad generally agreed with the previous reports of *igf1* mRNA expression levels in these tissues of medaka [25]. Similar to *Paralichthys olivaceus* [39], the *igf1r* involvement in reproduction and development was consistent with the functions of the *igf* system, such as *igf1* promoted spermatogenesis, oocyte maturation, and steroidogenesis [40,41], and *igf3* maintained the differentiation of ovary [42]. Furthermore, the expression of *igf1rs* mRNAs in the embryos at different developmental stages was demonstrated. Our present study further supports the potential functions of the *igf* system in fish.

In adult medaka, *igf1ra* and *igf1rb* mRNAs were detected in the oocytes at different stages; however, the expression levels were significantly different. During folliculogenesis, the level of *igf1ra* mRNA in mature follicles was extremely low in comparison with *igf1rb*, suggesting that *igf1ra* may play a minor role in mature follicles, while *igf1rb* plays a dominant role. Furthermore, through co-localization with the germ cell marker gene *vasa* [43], it was found that *igf1rb* RNA was highly expressed in many somatic cells, including theca cells and granulosa cells at later folliculogenesis, while *igf1ra* was absent. Similarly, *igf1r* was also found in theca cells and granulosa cells from the ovary of *Oncorhynchus kisutch* [44], gilthead seabream [9], alpaca [12], and mice [45]. Based on these findings, it indicates that the two *igf1rs* of medaka share some similar biological functions, which means that they play the same roles in many body activities. Meanwhile, it predicts that both of them also have their own unique roles in promoting individual growth and gonadal development. *Igf1rb* may also be involved in hormone production in the ovary of medaka according to previous studies that theca cells and granulosa cells regulate steroidal hormone production [44].

In this study, the two subtypes of *igf1r* were differentially expressed in the ovary as well as in the testis. Through the co-localization of *vasa* and *igf1rb*, as well as *igf1ra* and *igf1rb*, we found that both types of *igf1r* were expressed in sperm. In addition, *igf1rb* was also expressed in spermatogonia. Furthermore, it seemed that *igf1ra* preferred to express in Leydig cells, whereas *igf1rb* preferred to express in Sertoli cells, suggesting that the two isoforms may have different functions during spermatogenesis. These results agreed with the reported presence of *igf1* receptors in somatic cells of zebrafish [46], rainbow trout (*Oncorhynchus mykiss*) [13], and gilthead seabream [9]. In recent research on zebrafish, it has been found that *igf1rb* was expressed in spermatogonia and could mediate *igf3* to activate the β-catenin-dependent signal pathway to regulate spermatogenesis [46]. Moreover, it was shown that *igf1* and *igf2* interact with *igf1r*, causing the receptor activation and regulating organism growth, development, and reproduction [36]. Therefore, we speculate that the two subtypes of *igf1r* are essential for spermatogenesis, while *igf1rb* is also involved in the growth and proliferation of spermatogonia in the early stage. Overall, *igf1rs* play critical roles in the development of fish gonads, which is worthy of further research and provides a reference for other fish. Besides, the *igf* system is conserved in diverse species, and the interaction among ligands, receptors, and binding proteins of the system ensures that various life activities are carried out on the normal track, so the follow-up study of their co-localization is necessary for the further investigation of the *igf* system.

In summary, we demonstrated the differential expression of two *igf1r* subtypes in the adult gonads and embryos of medaka. The distinct expression patterns of the two subtypes of *igf1r* indicate that they play different roles in gonadal and embryonic development. Overall, the present study provides conclusive evidence for the potential roles of *igf1r* in gonadal development and gametogenesis in fish, as well as a reference for further research.

## Figures and Tables

**Table 1:** Sequences of primers used in the present study.

Primer	Sequence (5′ to 3′ Direction)	Purpose
*igf1ra*-F	ATGGACATCCGAAACGAC	RT-PCR
*igf1ra*-R	TCGTCTGTACAGGCCAGCTTG	
*igf1rb*-F	TGCTTCGGGAATGAGGCCTCC	RT-PCR
*igf1rb*-R	TGATGTGCAGGTTTCCTTTGAT	
*igf1ra*-F1	ATGGGCAGGCTGACCTTGTTTTGG	CDS cloning
*igf1ra*-R1	TTCGTCTGGAGTGTACATCTC	
*igf1ra*-F2	ATGTACACTCCAGACGAATGG	CDS cloning
*igf1ra*-R2	TCAGCAGGCCGACGACTGGGGCAG	
*igf1rb*-F1	ATGAGGCCTCCAGCGGAAACGAGG	CDS cloning
*igf1rb*-R1	GGCCACGCCCTCGTACACCAT	
*igf1rb*-F2	ATGGTGTACGAGGGCGTGGCC	CDS cloning
*igf1rb*-R2	CCCTTCAGCAGGCTGAG	
*igf1ra*-QF	CGCCTGCTTGGTGTAGTCT	Real-time PCR
*igf1ra*-QR	GGACCTGGCTGTTGGAGTT	
*igf1rb*-QF	GGTCTGATGCTGGCTCTGT	Real-time PCR
*igf1rb*-QR	ACTTCCTGGTTGGCGTTGT	
*Actin*-F	TTCAACAGCCCTGCCATGTA	Internal control
*Actin*-R	CCTCCAATCCAGACAGTAT	

## Data Availability

Not applicable.

## Supplementary Materials

The following supporting information can be downloaded at: https://www.mdpi.com/article/10.3390/life12060859/s1. Figure S1: Nucleotide and deduced amino acid sequence of medaka *igf1ra*; Figure S2: Nucleotide and deduced amino acid sequence of medaka *igf1rb*; Figure S3: Multiple sequence alignment of IGF1 receptors; Figure S4: Chromosome synteny diagrams for Igf1rs.

## Abbreviations

*Igf*, insulin-like growth factor; RT-PCR, reverse transcription-polymerase chain reaction; ISH, in situ hybridization; FISH, fluorescence in situ hybridization; P13K, phosphoinositide 3 kinase; aa, amino acid residues; nt, nucleotide.

## References

[1] Wood A.W., Duan C., Bern H.A. (2005). Insulin-like growth factor signaling in fish. Int. Rev. Cytol..

[2] Liu J.P., Baker J., Perkins A.S., Robertson E.J., Efstratiadis A. (1993). Mice carrying null mutations of the genes encoding insulin-like growth factor I (Igf-1) and type 1 IGF receptor (Igf1r). Cell.

[3] Berishvili G., D’Cotta H., Baroiller J.F., Segner H., Reinecke M. (2006). Differential expression of IGF-I mRNA and peptide in the male and female gonad during early development of a bony fish, the tilapia *Oreochromis niloticus*. Gen. Comp. Endocrinol..

[4] Moreira D.P., Melo R.M.C., Weber A.A., Rizzo E. (2020). Insulin-like growth factors 1 and 2 are associated with testicular germ cell proliferation and apoptosis during fish reproduction. Reprod. Fertil. Dev..

[5] Li J., Liu Z., Kang T., Li M., Wang D., Cheng C.H.K. (2021). Igf3: A novel player in fish reproductiondagger. Biol. Reprod..

[6] Li M., Liu X., Dai S., Xiao H., Qi S., Li Y., Zheng Q., Jie M., Cheng C.H.K., Wang D. (2020). Regulation of spermatogenesis and reproductive capacity by Igf3 in tilapia. Cell. Mol. Life Sci..

[7] Fan R., Ran M., Rui X., Jie M. (2021). m6A reader Igf2bp3 enables germ plasm assembly by m6A-dependent regulation of gene expression in zebrafish. Sci. Bull..

[8] Mei J., Yan W., Fang J., Yuan G., Chen N., He Y. (2014). Identification of a gonad-expression differential gene insulin-like growth factor-1 receptor (Igf1r) in the swamp eel (*Monopterus albus*). Fish Physiol. Biochem..

[9] Perrot V., Moiseeva E.B., Gozes Y., Chan S.J., Funkenstein B. (2000). Insulin-like growth factor receptors and their ligands in gonads of a hermaphroditic species, the gilthead seabream (*Sparus aurata*): Expression and cellular localization. Biol. Reprod..

[10] Kineman R.D., Del Rio-Moreno M., Sarmento-Cabral A. (2018). 40 YEARS of IGF1: Understanding the tissue-specific roles of IGF1/IGF1R in regulating metabolism using the Cre/loxP system. J. Mol. Endocrinol..

[11] Cannarella R., Condorelli R.A., La Vignera S., Bellucci C., Luca G., Calafiore R., Calogero A.E. (2020). IGF2 and IGF1R mRNAs are Detectable in Human Spermatozoa. World J. Mens Health.

[12] Gallelli M.F., Bianchi C., Lombardo D., Rey F., Rodriguez F.M., Castillo V.A., Miragaya M. (2019). Leptin and IGF1 receptors in alpaca (*Vicugna pacos*) ovaries. Anim. Reprod. Sci..

[13] Le Gac F., Loir M., le Bail P.Y., Ollitrault M. (1996). Insulin-like growth factor (IGF-I) mRNA and IGF-I receptor in trout testis and in isolated spermatogenic and Sertoli cells. Mol. Reprod. Dev..

[14] Schlueter P.J., Royer T., Farah M.H., Laser B., Chan S.J., Steiner D.F., Duan C. (2006). Gene duplication and functional divergence of the zebrafish insulin-like growth factor 1 receptors. FASEB J..

[15] Gu W., Yang Y., Ning C., Wang Y., Hu J., Zhang M., Kuang S., Sun Y., Li Y., Zhang Y. (2021). Identification and characteristics of insulin-like growth factor system in the brain, liver, and gonad during development of a seasonal breeding teleost, *Pampus argenteus*. Gen. Comp. Endocrinol..

[16] Maures T., Chan S.J., Xu B., Sun H., Ding J., Duan C. (2002). Structural, biochemical, and expression analysis of two distinct insulin-like growth factor I receptors and their ligands in zebrafish. Endocrinology.

[17] Schlueter P.J., Sang X., Duan C., Wood A.W. (2007). Insulin-like growth factor receptor 1b is required for zebrafish primordial germ cell migration and survival. Dev. Biol..

[18] Wittbrodt J., Shima A., Schartl M. (2002). Medaka—A model organism from the far East. Nat. Rev. Genet..

[19] Yi M., Hong N., Hong Y. (2009). Generation of medaka fish haploid embryonic stem cells. Science.

[20] Hong Y., Liu T., Zhao H., Xu H., Wang W., Liu R., Chen T., Deng J., Gui J. (2004). Establishment of a normal medakafish spermatogonial cell line capable of sperm production in vitro. Proc. Natl. Acad. Sci. USA.

[21] Hong N., Li M., Yuan Y., Wang T., Yi M., Xu H., Zeng H., Song J., Hong Y. (2016). Dnd is a critical specifier of primordial germ cells in the medaka fish. Stem Cell Rep..

[22] Li M., Hong N., Gui J., Hong Y. (2012). Medaka piwi is essential for primordial germ cell migration. Curr. Mol. Med..

[23] Matsuda M., Nagahama Y., Shinomiya A., Sato T., Matsuda C., Kobayashi T., Morrey C.E., Shibata N., Asakawa S., Shimizu N. (2002). DMY is a Y-specific DM-domain gene required for male development in the medaka fish. Nature.

[24] Nishimura T., Sato T., Yamamoto Y., Watakabe I., Ohkawa Y., Suyama M., Kobayashi S., Tanaka M. (2015). Sex determination. foxl3 is a germ cell-intrinsic factor involved in sperm-egg fate decision in medaka. Science.

[25] Yuan C., Chen K., Zhu Y., Yuan Y., Li M. (2018). Medaka igf1 identifies somatic cells and meiotic germ cells of both sexes. Gene.

[26] Yuan Y., Hong Y. (2017). Medaka insulin-like growth factor-2 supports self-renewal of the embryonic stem cell line and blastomeres in vitro. Sci. Rep..

[27] Xie J., Zhong Y., Zhao Y., Xie W., Guo J., Gui L., Li M. (2020). Characterization and expression analysis of gonad specific igf3 in the medaka ovary. Aquac. Fish..

[28] Iwamatsu T. (2004). Stages of normal development in the medaka *Oryzias latipes*. Mech. Dev..

[29] Tamura K., Stecher G., Peterson D., Filipski A., Kumar S. (2013). MEGA6: Molecular evolutionary genetics analysis version 6.0. Mol. Biol. Evol..

[30] Shved N., Berishvili G., D’Cotta H., Baroiller J.F., Segner H., Eppler E., Reinecke M. (2007). Ethinylestradiol differentially interferes with IGF-I in liver and extrahepatic sites during development of male and female bony fish. J. Endocrinol..

[31] Chen X., Zhu Y., Zhu T., Song P., Guo J., Zhong Y., Gui L., Li M. (2022). Vasa identifies germ cells in embryos and gonads of *Oryzias celebensis*. Gene.

[32] Song P., Sun B., Zhu Y., Zhong Y., Guo J., Gui L., Li M. (2021). Bucky ball induces primordial germ cell increase in medaka. Gene.

[33] Li M., Zhao H., Wei J., Zhang J., Hong Y. (2015). Medaka vasa gene has an exonic enhancer for germline expression. Gene.

[34] Schulz R.W., de Franca L.R., Lareyre J.J., Le Gac F., Chiarini-Garcia H., Nobrega R.H., Miura T. (2010). Spermatogenesis in fish. Gen. Comp. Endocrinol..

[35] Guo L., Yang S., Li M.M., Meng Z.N., Lin H.R. (2016). Divergence and polymorphism analysis of IGF1Ra and IGF1Rb from orange-spotted grouper, *Epinephelus coioides* (Hamilton). Genet. Mol. Res..

[36] Wu J., Li W., Craddock B.P., Foreman K.W., Mulvihill M.J., Ji Q.S., Miller W.T., Hubbard S.R. (2008). Small-molecule inhibition and activation-loop trans-phosphorylation of the IGF1 receptor. EMBO J..

[37] Hakuno F., Takahashi S.I. (2018). IGF1 receptor signaling pathways. J. Mol. Endocrinol..

[38] Kuang Y.M., Li W.S., Lin H.R. (2005). Molecular cloning and mRNA profile of insulin-like growth factor type 1 receptor in orange-spotted grouper, *Epinephelus coioides*. Acta Biochim. Biophys. Sin..

[39] Zhang J., Shi Z., Cheng Q., Chen X. (2011). Expression of insulin-like growth factor I receptors at mRNA and protein levels during metamorphosis of Japanese flounder (*Paralichthys olivaceus*). Gen. Comp. Endocrinol..

[40] Weber G.M., Sullivan C.V. (2000). Effects of insulin-like growth factor-I on in vitro final oocyte maturation and ovarian steroidogenesis in striped bass, *Morone saxatilis*. Biol. Reprod..

[41] Neirijnck Y., Calvel P., Kilcoyne K.R., Kuhne F., Stevant I., Griffeth R.J., Pitetti J.L., Andric S.A., Hu M.C., Pralong F. (2018). Insulin and IGF1 receptors are essential for the development and steroidogenic function of adult Leydig cells. FASEB J..

[42] Xie Y.X., Huang D., Chu L.H., Liu Y., Sun X., Li J.Z., Cheng C.H.K. (2021). Igf3 is essential for ovary differentiation in zebrafish. Biol. Reprod..

[43] Li M., Hong N., Xu H., Yi M., Li C., Gui J., Hong Y. (2009). Medaka vasa is required for migration but not survival of primordial germ cells. Mech. Dev..

[44] Maestro M.A., Planas J.V., Moriyama S., Gutierrez J., Planas J., Swanson P. (1997). Ovarian receptors for insulin and insulin-like growth factor I (IGF-I) and effects of IGF-I on steroid production by isolated follicular layers of the preovulatory coho salmon ovarian follicle. Gen. Comp. Endocrinol..

[45] Baumgarten S.C., Armouti M., Ko C., Stocco C. (2017). IGF1R expression in ovarian granulosa cells is essential for steroidogenesis, follicle survival, and fertility in female mice. Endocrinology.

[46] Safian D., Bogerd J., Schulz R.W. (2018). Igf3 activates beta-catenin signaling to stimulate spermatogonial differentiation in zebrafish. J. Endocrinol..

