# Supplementary material for: Differential Expression of Duplicate Insulin-like Growth Factor-1 Receptors (igf1rs) in Medaka Gonads
Source: Life (Basel). 2022 Jun 8;12(6):859. doi: 10.3390/life12060859 (PMC9225247; doi:10.3390/life12060859)
Supplement: Supplementary file 1 [file life-12-00859-s001.zip › life-1739754-supplementary.pdf]

```
1     ATGGGCAGGCTGACCTTGTTTTGGGGTCTGATGCTGTCTGTCTCCGCTGTCTGCATTTGCTCCACATATGGAGAGGTCTGTGAAAGCATGGACATCCGA
1      M  G  R  L  T  L  F  W  G  L  M  L  S  V  S  A  V  C  I  C  S  T  Y  G  E  V  C  E  S  M  D  I  R
100   AACGACATAAGTGAATTCAAGGTGCTGGAGAACTGCACGGTGGTTGAGGGTTACCTCCATATCCTCCTCATCAGCAAAAAAACCACCAACAACGTAGAG
34     N  D  I  S  E  F  K  V  L  E  N  C  T  V  V  E  G  Y  L  H  I  L  L  I  S  K  K  T  T  N  N  V  E
199   GCCTTACGTTCCCTCAGTTTCCCAAAGCTGACTGTCATCACTGACCATCTGCTGCTGTTTCGCGTTGCCGGCTTGGAGAGCCTCAGCACGCTGTTTCCC
67     A  L  R  S  L  S  F  P  K  L  T  V  I  T  D  H  L  L  L  F  R  V  A  G  L  E  S  L  S  T  L  F  P
298   AACCTGAGTGTCATCCGTGGTCGCAATCTGTTCTACAACTACGCCTTGGTGATTTACGAGATGACCAGCTTAAAGGACATAGGCTTGTACAACCTGAGA
100    N  L  S  V  I  R  G  R  N  L  F  Y  N  Y  A  L  V  I  Y  E  M  T  S  L  K  D  I  G  L  Y  N  L  R
397   AACATCACGCGCGGGGCTGTACGGGTTGCGAAGAACTCTGAACTTTGCTACCTGGACTCGGTGGACTGGTCTTTGATAACGGATGTTGTGTTCAACAAT
133    N  I  T  R  G  A  V  R  V  A  K  N  S  E  L  C  Y  L  D  S  V  D  W  S  L  I  T  D  V  V  F  N  N
496   GTTTTCGATTTGAATAAGAAATTGGAAGAGTGCGATAACGTCTGCCCAGGCATCATGAAGAATAGCCCTCTTTGTCCCAGGACGTCGTACACGAACAAG
166    V  F  D  L  N  K  K  L  E  E  C  D  N  V  C  P  G  I  M  K  N  S  P  L  C  P  R  T  S  Y  T  N  K
595   TTAGACTATCGCTGCTGGAGCTCCAACCATTGCCAGAAAGTGTGCCCAGATCAGTGCAAGCTGGCCTGTACAGACGACGGGCAGTGCTGCCACAGCCAG
199    L  D  Y  R  C  W  S  S  N  H  C  Q  K  V  C  P  D  Q  C  K  L  A  C  T  D  D  G  Q  C  C  H  S  Q
694   TGTTTGGGTGGCTGCAGCGAGCCCAACAGCGACCGGGCCTGCTCCGTCTGCCTCCACTATTTCCACAATGAGCGCTGCGTGCCAGACTGCCCACCAGGC
232    C  L  G  G  C  S  E  P  N  S  D  R  A  C  S  V  C  L  H  Y  F  H  N  E  R  C  V  P  D  C  P  P  G
793   ACGTACAAGTTTGAGGGCTGGCGCTGCATCACATTTGAGATGTGCTCCCAGGCGCACCTGCCAAGTGATACTCACTTTGTCATTCATGGGGGAGAATGT
265    T  Y  K  F  E  G  W  R  C  I  T  F  E  M  C  S  Q  A  H  L  P  S  D  T  H  F  V  I  H  G  G  E  C
892   ATGCCAGACTGCCCTTCTGATTTCACCCGCAACAAGACTAATCCCATGCTTTGTACTGCTTGCAACGGCCCGTGTGACAAGTTCTGCACTTCCCCTGTC
298    M  P  D  C  P  S  D  F  T  R  N  K  T  N  P  M  L  C  T  A  C  N  G  P  C  D  K  F  C  T  S  P  V
991   ATCGACTCTGTGGATGCTGCTCAAACCTTAAAGGGGTGCACCGTCATCAATGGCCATTTGGACATCAACATCCGCCGTGGAGATAACATAGCCTCTGAG
331    I  D  S  V  D  A  A  Q  T  L  K  G  C  T  V  I  N  G  H  L  D  I  N  I  R  R  G  D  N  I  A  S  E
1090  CTAGAGAACTTCATGGGCCTGATCCGGACAGTAAAGGGATATGTAAAGATACGACACTCCCATGCGCTCGGCTCCCTGTCCTTCCTCAGGAGTCTCCGA
364    L  E  N  F  M  G  L  I  R  T  V  K  G  Y  V  K  I  R  H  S  H  A  L  G  S  L  S  F  L  R  S  L  R
1189  TATATCAACGGGGAGGAACTTATTGAGAACACATATTCGTTCTTTGCCATCAACAACCAGCATCTGCAGTCCTTGTGGGACTGGAGTCAGCACAACTTG
397    Y  I  N  G  E  E  L  I  E  N  T  Y  S  F  F  A  I  N  N  Q  H  L  Q  S  L  W  D  W  S  Q  H  N  L
1288  ACAATCCGAGCTGGACGTCTCTTCTTCCGCCAGAACCCCAAGCTCTGCATGTCCGAGATCCACAAAATGTGGGAAAAGACGGGGATCACTGTGAAACCA
430    T  I  R  A  G  R  L  F  F  R  Q  N  P  K  L  C  M  S  E  I  H  K  M  W  E  K  T  G  I  T  V  K  P
1387  GAGGGGGGAGACTTCCGTAACAATGGAGAGAAAGCCAGCTGTGAAAGTCACATTCTGAAGATCAAATCTAACGTCACATCAAGTTATACAATCAAGCTG
463    E  G  G  D  F  R  N  N  G  E  K  A  S  C  E  S  H  I  L  K  I  K  S  N  V  T  S  S  Y  T  I  K  L
1486  ACATGGGAGCGCTATCAGGTTCCAGAATATGGAGGCCTGATCAACTTCATCGTTTTCTACAAAGAGTCACCTTTCCAAAACATCACAGAGTTTGATGGC
496    T  W  E  R  Y  Q  V  P  E  Y  G  G  L  I  N  F  I  V  F  Y  K  E  S  P  F  Q  N  I  T  E  F  D  G
1585  CAGGATGGCTGTGGCTCCAACAGCTGGCACATGGTGGATGTTGACCTTTCTCAGGATGAAAAACTTGACCCAAAGGTTACCCTCCAGCACCTCAATCCC
529    Q  D  G  C  G  S  N  S  W  H  M  V  D  V  D  L  S  Q  D  E  K  L  D  P  K  V  T  L  Q  H  L  N  P
1684  TGGACCCAGTACGCTATTTTTGTACGAGCAATCACCCTGCAGGTGGAGGACAAACACATCACCGGGGCAAAGAGTGACATCATTTACATCCGCACACGT
562    W  T  Q  Y  A  I  F  V  R  A  I  T  L  Q  V  E  D  K  H  I  T  G  A  K  S  D  I  I  Y  I  R  T  R
1783  CCATCATCGCCATCTGTGCCCAAAGATGCCCACGCCTATGCAAACTCCTCCACTAAGCTGCTGGTAAAGTGGTCGCGTCCGGTTTTTCCCAATGGCAAT
595    P  S  S  P  S  V  P  K  D  A  H  A  Y  A  N  S  S  T  K  L  L  V  K  W  S  R  P  V  F  P  N  G  N
1882  CTGACGTACTACCTGGTGCGCTGGCAGCAGCAGCCTGAAGACAGGGAGCTCTACCAGCATAACTACTGTTCCAAAGACCTGAAGATCCCCATTCGGGTG
628    L  T  Y  Y  L  V  R  W  Q  Q  Q  P  E  D  R  E  L  Y  Q  H  N  Y  C  S  K  D  L  K  I  P  I  R  V
1981  TCAGCTGGAGGATTCTCAGACGTGGAAGACGGCGCAAAACCCACCAAATCCGACCTTTCAGGTGCCAAGAAGGGTCCTTGCTGCACTTGCCAGAAGACA
661    S  A  G  G  F  S  D  V  E  D  G  A  K  P  T  K  S  D  L  S  G  A  K  K  G  P  C  C  T  C  Q  K  T
2080  GCTGAAGAGAAGGACCGTGAGAAGGACGACCGAGTCTTTATCAAAATGTTTGAGAACTACCTCCACAATATCGTCTTTTATGCAAGGCCTCCAGACCGT
694    A  E  E  K  D  R  E  K  D  D  R  V  F  I  K  M  F  E  N  Y  L  H  N  I  V  F  Y  A  R  P  P  D  R
2179  CAACGCAGGGACGTTGTTGGAGTCGCAAACAACACTTTGTTTCCGGATGGAGCTCAGGGAAATGCTTCGCTCAGTCCAGCGGAGAACGGCACAGATGGA
727    Q  R  R  D  V  V  G  V  A  N  N  T  L  F  P  D  G  A  Q  G  N  A  S  L  S  P  A  E  N  G  T  D  G
2278  GTTCCCCCCGTCATAGAGTACCCCTTCTCAGAGGAGAAGGTTTCCACAGAATACCTGGAGATCCCCAGCCTGCAACCTTTCACCGTCTACCGTATCGAC
760    V  P  P  V  I  E  Y  P  F  S  E  E  K  V  S  T  E  Y  L  E  I  P  S  L  Q  P  F  T  V  Y  R  I  D
2377  ATCCATGCCTGCAACGAGGAGGTGGGCCTCTGCGGTGCCGGAGCGTTCGTCTTCGCCAGGACCAAACCCGCATCTGAAGCAGATGACATCCCTGGAAAG
793    I  H  A  C  N  E  E  V  G  L  C  G  A  G  A  F  V  F  A  R  T  K  P  A  S  E  A  D  D  I  P  G  K
2476  GTCGTCCCTGAAAGGAGTGACAAGGTTGAGGGCTGTATGGTGCTGCACTGGCCAGAACCCGTTGTCCCCAATGGACTCGTACTGATGTATGAGGTCAAG
826    V  V  P  E  R  S  D  K  V  E  G  C  M  V  L  H  W  P  E  P  V  V  P  N  G  L  V  L  M  Y  E  V  K
2575  TACCGTCTGGGGACTGAGCCTGAGAAACACGGATGTGTGTCACGACGGCAATACCGGCAAAACAGAGGAGCTCGTCTGACCCACCTGAGCCCCGGGAAT
859    Y  R  L  G  T  E  P  E  K  H  G  C  V  S  R  R  Q  Y  R  Q  N  R  G  A  R  L  T  H  L  S  P  G  N
2674  TATTCAGCTCGTGTGCGGGCCATCTCTCTGGCCGGAAACGGATCCTGGACAGAGAGCATCTACTTTTACGTGCCTCCCCCTAAACGAGATGATGACGTT
892    Y  S  A  R  V  R  A  I  S  L  A  G  N  G  S  W  T  E  S  I  Y  F  Y  V  P  P  P  K  R  D  D  D  V
2773  ACGTTCTACTTGTCCATTATAATTCCCATTATAGTGACGCTCTTCATCGCCAGCCTCATCACCATTTTGTTCTTCATCAACAAAAAGAGGAACAGCAAC
925    T  F  Y  L  S  I  I  I  P  I  I  V  T  L  F  I  A  S  L  I  T  I  L  F  F  I  N  K  K  R  N  S  N
2872  AGACTGGGCAATGGAGTCCTGTATGCATCCGTCAACCCAGAGTACATAAGTGCAGCTGAGATGTACACTCCAGACGAATGGGAGGTGGCTCGAGAGAAG
958    R  L  G  N  G  V  L  Y  A  S  V  N  P  E  Y  I  S  A  A  E  M  Y  T  P  D  E  W  E  V  A  R  E  K
2971  ATCACCATGCACAAGGAGCTGGGTCAGGGCTCTTTTGGCATGGTGTACGAGGGCCTCGCAAAGGGCGTCGTCAAGGATGAACCTGAGACACGGGTGGCC
991    I  T  M  H  K  E  L  G  Q  G  S  F  G  M  V  Y  E  G  L  A  K  G  V  V  K  D  E  P  E  T  R  V  A
3070  ATCAAGACGGTCAATGAATCGGCCAGCATGAGGGAGCGGATCGAGTTTTTGAACGAGGCGTCAGTCATGAAGGAGTTCAACTGTCACCATGTGGTGCGC
1024   I  K  T  V  N  E  S  A  S  M  R  E  R  I  E  F  L  N  E  A  S  V  M  K  E  F  N  C  H  H  V  V  R
3169  CTGCTTGGTGTAGTCTCCCAGGGACAACCAACCTTGGTGATCATGGAGTTGATGACCCGGGGAGACCTCAAGAGCCACCTGCGCTCCCTACGCAACGAA
1057   L  L  G  V  V  S  Q  G  Q  P  T  L  V  I  M  E  L  M  T  R  G  D  L  K  S  H  L  R  S  L  R  N  E
3268  AACTCCAACAGCCAGGTCCTGCCACCGCTTAAAAAGATGATCCAGATGGCCGGAGAAATTGCAGACGGCATGGCCTACCTGAATGCCAATAAGTTTGTC
1090   N  S  N  S  Q  V  L  P  P  L  K  K  M  I  Q  M  A  G  E  I  A  D  G  M  A  Y  L  N  A  N  K  F  V
3367  CACAGGGACCTGGCTGCCAGGAACTGCATGGTTACAGAGGATTTCACAGTGAAGATTGGAGATTTCGGCATGACGAGAGATATTTATGAGACAGATTAT
1123   H  R  D  L  A  A  R  N  C  M  V  T  E  D  F  T  V  K  I  G  D  F  G  M  T  R  D  I  Y  E  T  D  Y
3466  TATCGAAAAGGGGGGAAAGGCCTGCTTCCTGTTCGCTGGATGTCTCCAGAGTCACTAAAAGATGGCGTGTTCACAACGATGTCTGACGTCTGGTCATTT
1156   Y  R  K  G  G  K  G  L  L  P  V  R  W  M  S  P  E  S  L  K  D  G  V  F  T  T  M  S  D  V  W  S  F
3565  GGCGTGGTGTTGTGGGAGATCGCTACGCTGTCGGAGCAGCCCTACCAGGGAATGTCCAATGAACAAGTGCTGCGCTTTGTCATGGAAGGAGGACTCCTG
1189   G  V  V  L  W  E  I  A  T  L  S  E  Q  P  Y  Q  G  M  S  N  E  Q  V  L  R  F  V  M  E  G  G  L  L
3664  GACAAACCCGACAACTGCCCTGACATGCTGTTTGAGCTAATGAGGATGTGCTGGCAGTATAACCCCAAGATGCGTCCGACCTTCCTGGAGATAATCAAC
1222   D  K  P  D  N  C  P  D  M  L  F  E  L  M  R  M  C  W  Q  Y  N  P  K  M  R  P  T  F  L  E  I  I  N
3763  ACCATCAAGGACGATCTGGATCCTTCCTTCAGGGAAGTCAGCTTCTTCTACAGCGAGGAGAACAAGCCGCCGGACACAGAGGAGCTGGATATGGAGGTA
1255   T  I  K  D  D  L  D  P  S  F  R  E  V  S  F  F  Y  S  E  E  N  K  P  P  D  T  E  E  L  D  M  E  V
3862  GAAAACATGGAGAACATTCCACTGGACCCCGTATCCACGAGGCAGCCGGCCGTTATTCACCCGTCCGGAAGCACACTGCCTCCTTCTGCCCAGCATTTG
1288   E  N  M  E  N  I  P  L  D  P  V  S  T  R  Q  P  A  V  I  H  P  S  G  S  T  L  P  P  S  A  Q  H  L
3961  GCCCCCATGCAAGGCCCAAGTACTCCTTTGCTGGGACCTGTGTCCCCCTCACACCCGGGCTATGTAGCTACAACTGTGGGATCTCCGGGACACGACAAG
1321   A  P  M  Q  G  P  S  T  P  L  L  G  P  V  S  P  S  H  P  G  Y  V  A  T  T  V  G  S  P  G  H  D  K
4060  CACTCAGGGCATGTTTTGGTCAACGGACCTGTGGTGGTGCTGCGGCCCAACTTTGAGGAGATCCAACCATATGCACACATGAACGGGGGCAGAAAGAAT
1354   H  S  G  H  V  L  V  N  G  P  V  V  V  L  R  P  N  F  E  E  I  Q  P  Y  A  H  M  N  G  G  R  K  N
4159  GAACGGGCATTACCGCTGCCCCAGTCGTCGGCCTGCTGA
1387   E  R  A  L  P  L  P  Q  S  S  A  C  *
```

Figure S1. Nucleotide and deduced amino acid sequence of medaka *igf1ra*. The putative translation initiation codons (underlined) located in the reading frame.

```
1    ATGAGGCCTCCAGCGGAAACGAGGCACTTGACCTTGTTTTGGGGTCTGATGCTGGCTCTGTCGTCCTGCCTCCGGCCCGCCACCGCAAAGATTTGTGGG
1     M  R  P  P  A  E  T  R  H  L  T  L  F  W  G  L  M  L  A  L  S  S  C  L  R  P  A  T  A  K  I  C  G
100  CCCAGCATCGACATTGGGAATGACATCAACGAATTCAGGCTTTTGGAGAACTGCACAGTGGTGGAGGGCTACCTGCAGATCCTCCTTATTGGCGACAAA
34    P  S  I  D  I  G  N  D  I  N  E  F  R  L  L  E  N  C  T  V  V  E  G  Y  L  Q  I  L  L  I  G  D  K
199  AATAACAACGCCAACCAGGAAGTCTTCCGCTCCCTCAGTTTCCCCAAGCTGACTATGATCACCGATTACCTGCTGCTATTCCGAGTGTCGGGCCTGGAC
67    N  N  N  A  N  Q  E  V  F  R  S  L  S  F  P  K  L  T  M  I  T  D  Y  L  L  L  F  R  V  S  G  L  D
298  AGTCTGAGCACGCTCTTTCCCAACCTCACTGTCATCCGGGGTCGAAATCTCTTCTACAACTACGCCCTGGTGATCTTTGAGATGACCAGCCTGAAAGAC
100   S  L  S  T  L  F  P  N  L  T  V  I  R  G  R  N  L  F  Y  N  Y  A  L  V  I  F  E  M  T  S  L  K  D
397  ATTGGGCTTTACAACCTGAGGAACATCACGCGTGGGGCCATCCGAATCGAGAAGAATCCAGAGCTCTGCTACCTTGACTCCATAGACTGGTCCTCCATC
133   I  G  L  Y  N  L  R  N  I  T  R  G  A  I  R  I  E  K  N  P  E  L  C  Y  L  D  S  I  D  W  S  S  I
496  ATGGATGCCGAGTTCAACAATTACATCGCTGGGAATAAGCAGTCCAAGGAGTGCAGCGACGTTTGCCTGGGCGTCACGGAGAACAACCCTCTTTGCAGA
166   M  D  A  E  F  N  N  Y  I  A  G  N  K  Q  S  K  E  C  S  D  V  C  L  G  V  T  E  N  N  P  L  C  R
595  AAGGCGATGATCCGGAACAGCTACAGCTACCACTGCTGGAACTCCAATCAGTGTCAGAAGGAGTGTCCACCAAGCTGTGGAAGGCGAGCTTGCACCGCT
199   K  A  M  I  R  N  S  Y  S  Y  H  C  W  N  S  N  Q  C  Q  K  E  C  P  P  S  C  G  R  R  A  C  T  A
694  GAGGGGAAGTGCTGCCATCCGCAGTGCCTGGGCAGCTGCACGGCTCCCGACAGCGACGCGGCGTGCGCGGCCTGCGTGCATTACTTCCACCAAGGGCGC
232   E  G  K  C  C  H  P  Q  C  L  G  S  C  T  A  P  D  S  D  A  A  C  A  A  C  V  H  Y  F  H  Q  G  R
793  TGCATGGCGGACTGCCCTCCAGGCACCTACCGGTTCGAGGGCTGGCGTTGCATCAGCGCCAAAGTCTGCTCTAAAGTCCACCACCCGGACTTTGACACC
265   C  M  A  D  C  P  P  G  T  Y  R  F  E  G  W  R  C  I  S  A  K  V  C  S  K  V  H  H  P  D  F  D  T
892  TTCGTCATCCACGGGGGAGAGTGCATGCCTGAGTGTCCGTCCGGTTACACCCGCTCTGCCACCAACAGCATGCACTGCACAGCCTGTGATGGTCTGTGC
298   F  V  I  H  G  G  E  C  M  P  E  C  P  S  G  Y  T  R  S  A  T  N  S  M  H  C  T  A  C  D  G  L  C
991  GACAAAGTCTGCGACGGGAAGGTCATCGACTCCATGGATGCCGCCCAGTCGCTCAAAGGCTGCACCGTGATCAAAGGAAACCTGCACATCAACATCCGC
331   D  K  V  C  D  G  K  V  I  D  S  M  D  A  A  Q  S  L  K  G  C  T  V  I  K  G  N  L  H  I  N  I  R
1090 AGAGGCCACAACATGGTGGCCGAGCTGGAGAGCTTCACGGGTCTGATCCAGAGGGTGACGGGTTACGTTCGGATCACCCACTCCCACACACTGACCTCC
364   R  G  H  N  M  V  A  E  L  E  S  F  T  G  L  I  Q  R  V  T  G  Y  V  R  I  T  H  S  H  T  L  T  S
1189 CTGGCTTTCCTGCGCAGCCTCAGATACATCGACGGAGAGACCCTGCTGGAAGACATGTACGCCTTCTCGGCGTTCGACAACCAGCAGCTCCAGTACCTC
397   L  A  F  L  R  S  L  R  Y  I  D  G  E  T  L  L  E  D  M  Y  A  F  S  A  F  D  N  Q  Q  L  Q  Y  L
1288 TGGGACTGGAAGCAGCACAACCTCACCATAAAAACAGGAAAGCTGTTCTTCAGAGCCAACCCCAAGCTCTGCATGTCCGAGATCCGCAAGATGTGGACC
430   W  D  W  K  Q  H  N  L  T  I  K  T  G  K  L  F  F  R  A  N  P  K  L  C  M  S  E  I  R  K  M  W  T
1387 AAGACGGGCATCCGGGAGCATTTTCAGGAGACAGACTTCAGAAACAACGGCGACAGAGCCAGCTGTGAAAGCACAATCCTGCGCTTCAAGTCCAACAGC
463   K  T  G  I  R  E  H  F  Q  E  T  D  F  R  N  N  G  D  R  A  S  C  E  S  T  I  L  R  F  K  S  N  S
1486 ACCCAGAGCACCAGGATCATCCTGACATGGGAGCGCTACCGGCCCCCCGACTACAGAGACCTCATCAGCTTCATCGTGTACTACAAGGAGGCACCCTTT
496   T  Q  S  T  R  I  I  L  T  W  E  R  Y  R  P  P  D  Y  R  D  L  I  S  F  I  V  Y  Y  K  E  A  P  F
1585 CAGAACATCACAGAGTTCGAGGGGCAGGACGGCTGCGGCTCCAACAGCTGGAACATGGTGGACGTGGAGCTGCGGCCCGAGAAGGACTCGGACCCCGGG
529   Q  N  I  T  E  F  E  G  Q  D  G  C  G  S  N  S  W  N  M  V  D  V  E  L  R  P  E  K  D  S  D  P  G
1684 GTTCTGCTGTCCGGCCTGAAACCCTGGACGCAGTACGCCATCTTCGTCAAAGCCGTCACCCTCATGGTGGAGGACAAGTTCATGCCCGGAGCCAAGAGC
562   V  L  L  S  G  L  K  P  W  T  Q  Y  A  I  F  V  K  A  V  T  L  M  V  E  D  K  F  M  P  G  A  K  S
178  AAAGTGGTGTACATCCGCACCAGCCCTTCACCACCCACCATGCCCCGGGACGTGCGGGCGTACTCCAACTCCTCCACCCAGCTGGTGGTCCGCTGGTTG
595   K  V  V  Y  I  R  T  S  P  S  P  P  T  M  P  R  D  V  R  A  Y  S  N  S  S  T  Q  L  V  V  R  W  L
1882 CCCCCCGTCTCACCCAACGGGAACCGGACATACTACCTCATAAGGTGGCAACAACAGGCGGAGGACCGCGAGCTGTTCCAGCACAACTACTGCTCCAAA
628   P  P  V  S  P  N  G  N  R  T  Y  Y  L  I  R  W  Q  Q  Q  A  E  D  R  E  L  F  Q  H  N  Y  C  S  K
1981 GCAGAGCTGAAGATCCCTGTGAGGATCGCTGCCAGAGGCGTCGGAGACCAAGAAGAGGAGACGAAACCCACCAAATCCGATACCGAGGGGGCAAATAAA
661   A  E  L  K  I  P  V  R  I  A  A  R  G  V  G  D  Q  E  E  E  T  K  P  T  K  S  D  T  E  G  A  N  K
2080 GGCCCGTGCTGCCTCTGCCCCAAATCCCCAGAAGTCCTGGAGGCTGAAGCTGCTGACGCCTCCTACAGAAAAGTCTTTGAGAATTTCCTGCACAATTCT
694   G  P  C  C  L  C  P  K  S  P  E  V  L  E  A  E  A  A  D  A  S  Y  R  K  V  F  E  N  F  L  H  N  S
2179 ATCTTTACGCCAAGCTTTAGGCCGGCTGATCGGCGCCGAAGAGACGTCTTTGGTATCGCCAACTCCACATACGCGGGGCGCCACAAGTCCTTAAGCAAC
727   I  F  T  P  S  F  R  P  A  D  R  R  R  R  D  V  F  G  I  A  N  S  T  Y  A  G  R  H  K  S  L  S  N
2278 ACCAGCGCCGACCCGCCTCCCCACACAGCCGGAAACAGCAGCGGCGCCGCCGAACCGGAGCCGGCCGACCAAGAGTACGAGTTCCTGGAGCAGTCCGTG
760   T  S  A  D  P  P  P  H  T  A  G  N  S  S  G  A  A  E  P  E  P  A  D  Q  E  Y  E  F  L  E  Q  S  V
2377 ACGGAGCGTGAGCTGCAGATCTCCGGCCTCCAGCCCTTCACCGTTTACCGCGTCGACGTTCACGCCTGCAACCGACAAGTCAAGGAGTGCAGCGCCGCC
793   T  E  R  E  L  Q  I  S  G  L  Q  P  F  T  V  Y  R  V  D  V  H  A  C  N  R  Q  V  K  E  C  S  A  A
2476 GAGTTTGTGTTCTCCAGAACCAAACCAGCCGAAAAGGCCGATGACGTACCAGGCCCGGTGACCTGGGAGGGCCACAAAGACTGGGTGTTTCTGCGCTGG
826   E  F  V  F  S  R  T  K  P  A  E  K  A  D  D  V  P  G  P  V  T  W  E  G  H  K  D  W  V  F  L  R  W
2575 CCTGAGCCGTCCCATCCCAACGGGCTTATCCTCCTCTACGAGATCAAGTACAAACTGGCTGCAGAGAGCGAGAAACACGAGTGCGTGTCCAGACAGCTC
859   P  E  P  S  H  P  N  G  L  I  L  L  Y  E  I  K  Y  K  L  A  A  E  S  E  K  H  E  C  V  S  R  Q  L
2674 TACCAGACCCAGCAGGGCGTTCGGCTGACCAACCTCAGTCCAGGAAACTATTCAGTCAGAGTGAGAGCCACGTCACTGGCCGGCAACGGCTCCTGGACG
892   Y  Q  T  Q  Q  G  V  R  L  T  N  L  S  P  G  N  Y  S  V  R  V  R  A  T  S  L  A  G  N  G  S  W  T
2773 CCACCGCTGGACTTCTACGTGGCAGAAAGATACGAAAGCGCGCTGTACGCCATGGTCTTCGTTCCCATCATCATCGTCATCCTGATCAGCTGCATGGTC
925   P  P  L  D  F  Y  V  A  E  R  Y  E  S  A  L  Y  A  M  V  F  V  P  I  I  I  V  I  L  I  S  C  M  V
2872 GTCATGTTGGTCGCCCTCAACAGGAAAAGGGAGGTCGACCGTTGCACCTCTGGCCCCACAGCTGCAAACCCACAAAAGTGTATCAGTAGAAACTCCTGC
958   V  M  L  V  A  L  N  R  K  R  E  V  D  R  C  T  S  G  P  T  A  A  N  P  Q  K  C  I  S  R  N  S  C
2971 TGTAAATGGATTTTTTCAATTTTTCGTTCCAAAACCTGTCGTTGCTATGTTTTATCGCAGCAGGGGTCGTTCGGCATGGTGTACGAGGGCGTGGCCAAA
991   C  K  W  I  F  S  I  F  R  S  K  T  C  R  C  Y  V  L  S  Q  Q  G  S  F  G  M  V  Y  E  G  V  A  K
3070 GGCGTGGTCAAAGACGAACCGGAGACCAAGGTCGCCGTCAAGACGGTTAACGAGTCGGCCAGCATGCGGGAGAGGATCGAGTTCCTCAACGAGGCGTCG
1024  G  V  V  K  D  E  P  E  T  K  V  A  V  K  T  V  N  E  S  A  S  M  R  E  R  I  E  F  L  N  E  A  S
3169 GTCATGAAGGAGTTCAACTGTCACCATGTGGTTCGACTCCTGGGTGTGGTCTCCCAGGGCCAGCCCACCCTGGTCATCATGGAGCTGATGACGAGAGGA
1057  V  M  K  E  F  N  C  H  H  V  V  R  L  L  G  V  V  S  Q  G  Q  P  T  L  V  I  M  E  L  M  T  R  G
3268 GACCTGAAGAGTTACCTGCGCTCGCTGCGACCTAAAGAGCAACAGTGGTCCAGCCTGTCTCTTCCTCCTCTGAAGAAGATGCTTCAGATGGCGGGGCAG
1090  D  L  K  S  Y  L  R  S  L  R  P  K  E  Q  Q  W  S  S  L  S  L  P  P  L  K  K  M  L  Q  M  A  G  Q
3367 ATCGCTGACGGCATGGCGTACCTCAACTCCAACAAGTTTGTCCACCGAGACCTGGCAGCCAGGAACTGCATGGTGGCCGAAGACTTTACCGTTAAAATC
1123  I  A  D  G  M  A  Y  L  N  S  N  K  F  V  H  R  D  L  A  A  R  N  C  M  V  A  E  D  F  T  V  K  I
3466 GGAGACTTTGGCATGACCAGAGACATCTATGAGACGGATTACTACCGTAAAGGGGGCAAAGGTTTGCTCCCCGTCCGCTGGATGTCGCCCGAGTCTCTG
1156  G  D  F  G  M  T  R  D  I  Y  E  T  D  Y  Y  R  K  G  G  K  G  L  L  P  V  R  W  M  S  P  E  S  L
3565 AAGGACGGCGTCTTCACCACCAACTCCGATGTCTGGTCGTTTGGAGTTGTGTTGTGGGAGATCGCCACCCTGGCAGAGCAGCCGTACCAGGGTCTGTCC
1189  K  D  G  V  F  T  T  N  S  D  V  W  S  F  G  V  V  L  W  E  I  A  T  L  A  E  Q  P  Y  Q  G  L  S
3664 AACGAGCAAGTGCTCCGCTTTGTCATGGAGGGGGGGCTGCTGGAAAAGCCGCAGAACTGTCCCGACATGCTGTTCGAGCTGATGCGAATGTGCTGGCAG
1222  N  E  Q  V  L  R  F  V  M  E  G  G  L  L  E  K  P  Q  N  C  P  D  M  L  F  E  L  M  R  M  C  W  Q
3763 TACAATCCTAAAATGCGTCCGTCCTTCGTGGAAATCATCGGCAGCGTAAAGGACGAGCTCGACCCGTCTTTCAAAGAGGTCAGCTTCTATTACAGCGCC
1255  Y  N  P  K  M  R  P  S  F  V  E  I  I  G  S  V  K  D  E  L  D  P  S  F  K  E  V  S  F  Y  Y  S  A
3862 GACAACAAGCCCGCCGAGGCCTCGCAGCTCCACCCTGACAAGGTGGAGGCCCCAGAGGACGCCCCAGAGGACCCCCAGACCCCCCAGCAGACGCCTCCA
1288  D  N  K  P  A  E  A  S  Q  L  H  P  D  K  V  E  A  P  E  D  A  P  E  D  P  Q  T  P  Q  Q  T  P  P
3961 TCGCCCAGCTCGGAGGTCCTGCCAGCCTCCCCGCTGGCCCAAAGCTCTCTTTCATCGCCGTGCACGTCGAGCACGGTCACGGACAAGCAGCCCCCGGGC
1321  S  P  S  S  E  V  L  P  A  S  P  L  A  Q  S  S  L  S  S  P  C  T  S  S  T  V  T  D  K  Q  P  P  G
4060 CAGCAGGCGGCCAATGGGCTCTCGGGGGCGGGCCTTCCGGCGGCGTCGGCCGGCTCGAGCGTCACACTTCGGCCTTCTCTGGACGAACTGCCTCCGTAT
1354  Q  Q  A  A  N  G  L  S  G  A  G  L  P  A  A  S  A  G  S  S  V  T  L  R  P  S  L  D  E  L  P  P  Y
4159 GCTCACATGAACGGAGGGCGCAAAAACGAGCGTGCCATGCCACTCCCACAATCCTCAGCCTGCTGA
1387  A  H  M  N  G  G  R  K  N  E  R  A  M  P  L  P  Q  S  S  A  C  *
```

Figure S2. Nucleotide and deduced amino acid sequence of medaka *igf1rb*. The putative translation initiation codons (underlined) located in the reading frame.

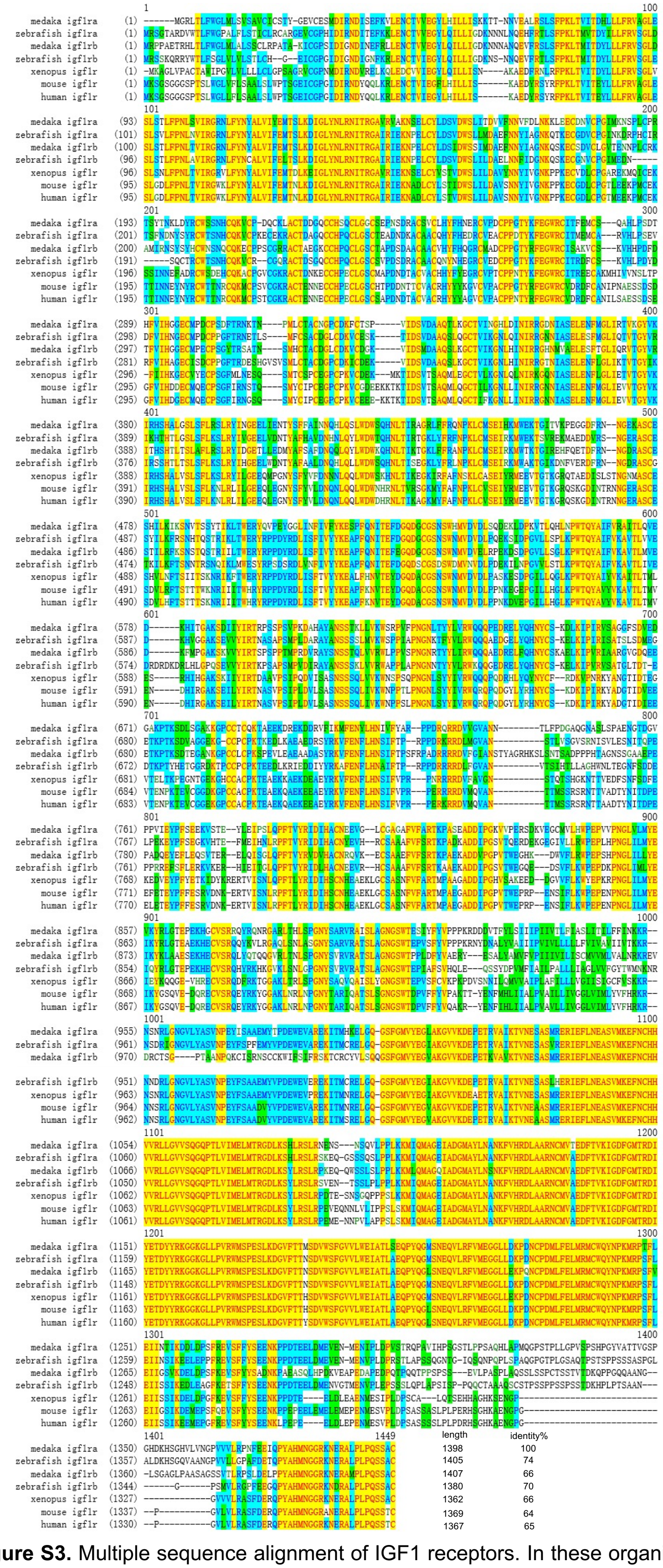

Figure S3. Multiple sequence alignment of IGF1 receptors. In these organisms, Igf1r was used as the ortholog for comparison for both medaka Igf1ra and Igf1rb as the existence of paralogous isoforms of the IGF1R in mammals and amphibians.

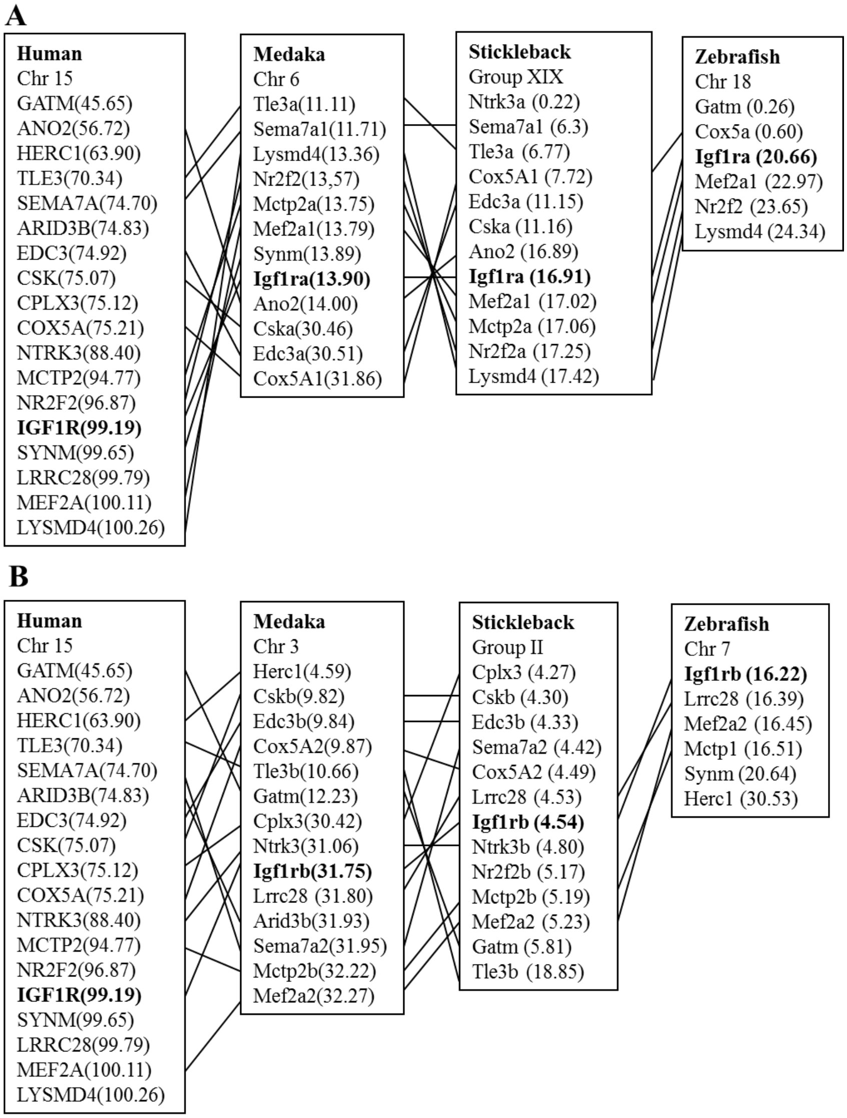

Figure S4. Chromosome synteny diagrams for Igf1rs. (A) Synteny for Igf1ra. (B) Synteny for Igf1rb. Chr, chromosome. Numbers in parentheses correspond to the position on the chromosome in Mb.
